# Effects of treatment of non-alcoholic fatty liver disease on heart failure with preserved ejection fraction

**DOI:** 10.3389/fcvm.2022.1120085

**Published:** 2023-01-12

**Authors:** Zifeng Yang, Ruifeng Tian, Xiao-Jing Zhang, Jingjing Cai, Zhi-Gang She, Hongliang Li

**Affiliations:** ^1^Department of Cardiology, Renmin Hospital of Wuhan University, Wuhan, China; ^2^Institute of Model Animal, Wuhan University, Wuhan, China; ^3^School of Basic Medical Sciences, Wuhan University, Wuhan, China; ^4^Department of Cardiology, The Third Xiangya Hospital, Central South University, Changsha, China; ^5^Gannan Innovation and Translational Medicine Research Institute, Gannan Medical University, Ganzhou, China

**Keywords:** nonalcoholic fatty liver disease, heart failure with preserved ejection fraction, risk factor, systemic inflammation, secretory factors, epicardial adipose tissue, treatment

## Abstract

In the past few decades, non-alcoholic fatty liver disease (NAFLD) and heart failure with preserved ejection fraction (HFpEF) have become the most common chronic liver disease and the main form of heart failure (HF), respectively. NAFLD is closely associated with HFpEF by sharing common risk factors and/or by boosting systemic inflammation, releasing other secretory factors, and having an expansion of epicardial adipose tissue (EAT). Therefore, the treatments of NAFLD may also affect the development and prognosis of HFpEF. However, no specific drugs for NAFLD have been approved by the Food and Drug Administration (FDA) and some non-specific treatments for NAFLD are applied in the clinic. Currently, the treatments of NAFLD can be divided into non-pharmacological and pharmacological treatments. Non-pharmacological treatments mainly include dietary intervention, weight loss by exercise, caloric restriction, and bariatric surgery. Pharmacological treatments mainly include administering statins, thiazolidinediones, glucagon-like peptide-1 receptor agonists, sodium-glucose cotransporter 2 inhibitors, and metformin. This review will mainly focus on analyzing how these treatments may affect the development and prognosis of HFpEF.

## 1. Introduction

Non-alcoholic fatty liver disease (NAFLD) is one of the most prevalent chronic liver diseases without specific treatments and covers the disease spectrum from simple steatosis to non-alcoholic steatohepatitis (NASH), which can evolve into end-stage liver diseases (1–3). With a global incidence of 25.24%, NAFLD brings up a huge clinical and economic burden (2). The clinical and economic burden of NAFLD arises not only from liver-related morbidity and mortality but also from extrahepatic diseases, such as type 2 diabetes mellitus (T2DM) and cardiovascular disease (CVD) (4). Heart failure with preserved ejection fraction (HFpEF) is one of the major CVDs associated with NAFLD (5, 6). HFpEF is the most common type of heart failure (HF) and even has an incidence rate of 55% among the elderly (7, 8). A recent clinical study demonstrated that individuals with NAFLD are at increased risk of developing HF and that NAFLD is more likely to coexist with HFpEF (9). In addition, the coexistence of NAFLD and HFpEF often occurs in patients with HFpEF, and the prevalence of NAFLD in patients with HFpEF can reach 50% (10). All these facts indicate the tight association between NAFLD and HFpEF.

Theoretically, NAFLD is associated with HFpEF in two layers. On the one hand, NAFLD and HFpEF share common risk factors. Several documented risk factors of NAFLD, including age, male sex, obesity, and T2DM (11–13), are also known risk factors for HFpEF (14–16). On the other hand, accumulating compelling evidence indicates that the pathological consequence of NAFLD may promote the development of HFpEF. It is well known that NAFLD readily boosts systemic inflammation and has thicker epicardial adipose tissue (EAT) (17–19). In addition, a large number of other secretory factors are also released in NAFLD (20). These pathological processes play crucial roles in the progression of HFpEF (21–23). The close association between NAFLD and HFpEF indicates that treating NAFLD may simultaneously affect the progression of HFpEF.

However, there are no specific drugs approved for the treatment of NAFLD yet, management of NAFLD is currently achieved by employing non-specific non-pharmacological and/or pharmacological treatments. Non-pharmacological treatments mainly include dietary intervention, weight loss by exercise, caloric restriction, and bariatric surgery. Pharmacological treatments mainly include administering statins, thiazolidinediones, glucagon-like peptide-1 receptor agonists, sodium-glucose cotransporter 2 inhibitors, and metformin. These non-pharmacological and pharmacological treatments can effectively treat the liver in NAFLD/NASH patients (24–26). Interestingly, extensive literature shows that some treatments, which are commonly used in the treatment of NAFLD/NASH, coincidentally have a beneficial or detrimental impact on decreasing the effects of secretory factors, or reducing EAT accumulation, controlling common risk factors for NAFLD and HFpEF (27–29). Therefore, it can be speculated that some treatments for NAFLD may also affect the development of HFpEF.

This review mainly focuses on overviewing the possible effects of current interventions available for NAFLD on the development and prognosis of HFpEF.

## 2. NAFLD is closely associated with HFpEF

**FIGURE 1:**
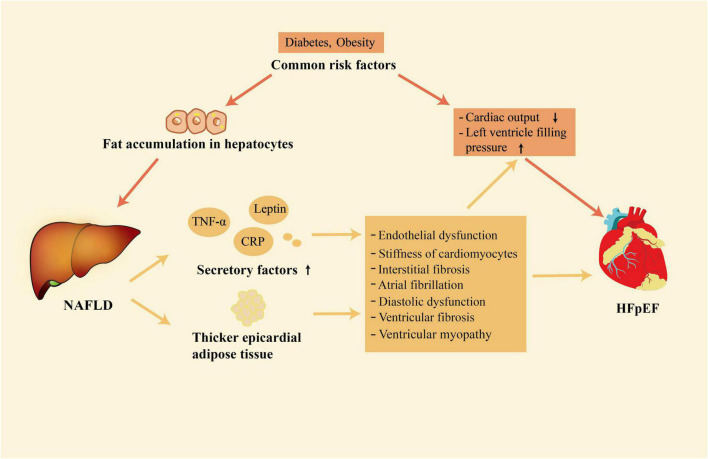
The close association between NAFLD and HFpEF. NAFLD and HFpEF have some common risk factors; in addition, NAFLD is associated with the development of HFpEF through its association with release of secretory factors and increased accumulation of epicardial adipose tissue. NAFLD, non-alcoholic fatty liver disease; HFpEF, heart failure with preserved ejection fraction.

NAFLD and HFpEF are both systemic diseases (4, 30). Growing evidence shows that there is a close relationship between NAFLD and HFpEF (6, 9, 10). This association mainly exists in two layers. Firstly, NAFLD and HFpEF share common risk factors. Secondly, NAFLD may contribute to the development of HFpEF by enhancing systemic inflammation, releasing of other secretory factors, and having thicker EAT (Figure 1).

### 2.1. NAFLD and HFpEF share common risk factors

NAFLD is characterized by excessive fat accumulation in hepatocytes without or with lobular inflammation and fibrosis and affects more than a billion people worldwide today (31, 32). NAFLD covers several liver diseases ranging from hepatic steatosis to NASH. Simple hepatic steatosis has been defined as NAFL, and NASH has been defined as a serious subtype of NAFLD with a more severe process of hepatocellular injury and inflammation (33). Moreover, NASH is often accompanied by liver fibrosis and even progresses to cirrhosis. Based on imaging, some clinical features such as elevated liver enzymes and the presence of metabolic comorbidities, or histology, NAFLD can be diagnosed in individuals (34). There are many risk factors for NAFLD, including obesity, and T2DM (35, 36). Together with NAFLD, these risk factors constitute major and causative risk factors of metabolic diseases and are mediators of the occurrence and development of CVDs, including HFpEF (37–40). Exposure to these risk factors, especially obesity and T2DM, contributes to an increased risk of NAFLD and HFpEF in the population (41, 42). More importantly, because they share risk factors, NAFLD and HFpEF are likely to coexist in the same individuals (10). These common risk factors should be one of the key mechanisms underlying the epidemiological link between NAFLD and HFpEF.

### 2.2. NAFLD is associated with the development of HFpEF

HFpEF has been defined as a multisystem disorder with multiple pathophysiological abnormalities. In the pathophysiology of HFpEF, left ventricular diastolic dysfunction plays a crucial and indispensable role (43). In addition to an abnormality in left ventricular diastolic function, HFpEF is a complex syndrome that has multiple injuries of cardiac reserve and rhythm, endothelial dysfunction, atrial dysfunction, chronotropic reserve deficits, ventricular and vascular stiffness, defects in vasodilation, pulmonary hypertension, peripheral microvascular dysfunction (43, 44). Coincidentally, NAFLD is associated with abnormalities in cardiac structure and function, which are common in HFpEF (45–47). NAFLD may be associated with the development of HFpEF through enhancing systemic inflammation, releasing of other secretory factors, and having thicker EAT (21–23, 48).

During the pathogenies of NAFLD, increased amounts of lipids accumulate in the liver and lead to oxidative stress, cell death of hepatocytes, recruitment of inflammatory cells, and increased secretion of inflammatory factors (49). The immune cell population of the liver such as Kupffer cells, dendritic cells, and macrophages switches from an immune tolerance phenotype to an immunogenic phenotype in the pathogenesis of NAFLD (50). The shift of proinflammatory phenotype and the activation of the immune response promote the production and release of cytokines, increasing the penetration of circulating immune cells into the liver and enhancing local inflammatory response (51). Then a large number of inflammatory mediators are carried by the blood from the hepatic sinus into the systemic circulation (52). The increased amount of inflammatory molecule secretion, including tumor necrosis factor-A (TNF-α), C-reactive protein (CRP), interleukin-6 (IL-6), and some other pro-inflammations (53), boosts systemic inflammation. Systemic inflammation not only contributes to the transition from simple steatosis into NASH but also poses a threat to the cardiovascular system (21). It has been reported that systemic inflammation would induce coronary microvascular endothelial dysfunction (22). Then, the bioavailability of nitric oxide and the activity of protein kinase G in adjacent cardiomyocytes are decreased by coronary microvascular endothelial inflammation (54). Low activity of protein kinase G contributes to elevated resting tension and cardiac hypertrophy, resulting in interstitial fibrosis and cardiomyocyte stiffness (22, 55, 56). Both interstitial fibrosis and stiff cardiomyocytes promote left ventricular stiffness and the development of HFpEF. A meta-analysis showed that high circulating levels of inflammatory markers, such as CRP and IL-6, also induce atrial fibrillation in the general population (57). Atrial fibrillation is not only closely associated with diastolic dysfunction, but also an important risk factor of HFpEF, which can predict HFpEF (58, 59). Meanwhile, elevated levels of inflammatory markers themselves, such as CRP, are independently associated with asymptomatic diastolic dysfunction (60).

Inflammatory factors play a key role in linking NAFLD and HFpEF. Meanwhile, NAFLD could also promote the development of HFpEF through the effects of other secretory factors such as angiotensinogen, leptin, and hepatokines. Angiotensinogen is mainly secreted by the liver and is the only precursor of the renin-angiotensin system (61). In the case of obesity, the adipose tissue can also produce a large amount of angiotensinogen and lead to elevation of the circulating angiotensinogen (62), which is pretty common in patients with NAFLD. Increased circulating angiotensinogen levels lead to excessive production of angiotensin I and angiotensin II (63). Eventually, this scenario leads to the activation of the renin-angiotensin system and increases the risk of CVD, including HFpEF. In patients with NAFLD, the sensitivity to leptin is usually reduced, leading to an increase in the levels of leptin (64). High leptin levels can lead to ventricular fibrosis and cardiomyocyte hypertrophy (65). At the same time, leptin is also associated with sodium retention and increased plasma volume, which would increase the risk of heart failure (66). The liver also secretes a class of proteins called hepatokines. Some cardiovascular risk-related hepatokines, such as fetuin-A and fibroblast growth factor-21, are increased in NAFLD (67, 68). Fetuin-A can increase the proliferation of smooth muscle cells and the deposition of collagen, thus accelerating vascular remodeling and resulting in the occurrence of HFpEF (69). Fibroblast growth factor-21, however, exerts an anti-inflammation function, and inflammation plays a key role in the development of HFpEF (70). Therefore, the net effects of hepatokines on the cardiovascular system in the case of NAFLD are still unclear and need to be further explored.

In addition to systemic inflammation and the effects of other secretory factors, EAT also plays a significant role in the association between NAFLD and HFpEF. In addition to energy storage, adipose tissue is also an endocrine organ that can secrete many proinflammatory adipokines in chronic systemic disorders (71). Compared with normal subjects, EAT is increased in individuals with NAFLD and the degree of expansion is related to the severity of liver steatosis and fibrosis (72). The expansion of EAT is often accompanied by the upregulation of proinflammatory cytokines and infiltration of immune cells such as macrophages, dendritic cells, and T lymphocytes. Consequently, EAT has become an important source of proinflammatory and profibrotic mediators such as IL-6, monocyte chemoattractant protein-1, TNF-α, and interleukin-1β (73). These mediators can cause ventricular fibrosis and coronary microvascular dysfunction, leading to the development of HFpEF (71, 74, 75). It was reported that adjoin of dysfunctional EAT with the left ventricle resulted in ventricular myopathy characterized by impaired cardiac dilatation, which would lead to HFpEF (76). Conversely, removing EAT can improve the structure of vascular tissue and restore diastolic function (77, 78).

NAFLD may also be associated with the development of HFpEF through other pathophysiological processes such as metabolic abnormalities and oxidative stress. The overproduction of glucose and triglycerides in NAFLD would cause hyperglycemia and hyperglyceridemia (79). Hyperglycemia and hyperglyceridemia are the key features of the pathophysiology of metabolic syndrome. Furthermore, excessive fat accumulation in hepatocytes induces oxidative stress and the overproduction of reactive oxygen species in the mitochondria (80). Both metabolic syndrome and oxidative stress are closely related to the development of HFpEF (81, 82).

In summary, accumulating studies have indicated that a close association is between NAFLD and HFpEF. Therefore, the treatments against NAFLD are also likely to exert beneficial or harmful effects on the development and prognosis of HFpEF.

### 2.3. NAFLD directly leads to the occurrence of HFpEF

The above evidence indicates the close association between NAFLD and HFpEF. Moreover, accumulating studies also show that the progression of NAFLD may result in the occurrence of HFpEF, probably by preventing blood flow back to the heart, inducing changes in hemodynamics, and releasing secretory factors.

About 25% of the total blood returns to the heart through the liver (83). When liver fibrosis progressively deteriorates, it would increase resistance in the hepatic sinus and lead to obstruction of blood flow through the liver (84, 85). This blockage prevents the return of blood from the veins to the heart. Some studies have shown that this phenomenon even existed in the early stage of NAFLD (84). During the development of NAFLD, the liver changes from simple hepatic steatosis to NASH, and eventually to cirrhosis (86). Portal hypertension is one of the typical characteristics of liver cirrhosis (87). Thus, spontaneous portosystemic shunt and arteriovenous shunt would be formed in the late stage of NAFLD, resulting in hemodynamic changes with increased cardiac output at rest (6, 88). These alterations limit the preload reserve of the heart and make it difficult to increase cardiac output during stress, eventually resulting in the occurrence of HFpEF. Finally, during the progression of NAFLD, many secretory factors would be increased in circulation, leading to the occurrence of HFpEF, as stated in section 2.2.

In summary, NAFLD can lead to the occurrence of HFpEF through the above-mentioned mechanism. In this aspect, more direct evidence is needed to confirm the causal relationship and to facilitate discovering the detailed molecular pathways. Employing the power of emerging frontier technologies, such as multi-omics technologies and single-cell technologies, may help us to achieve these goals.

## 3. Treatments for NAFLD and their impact on HFpEF

**FIGURE 2:**
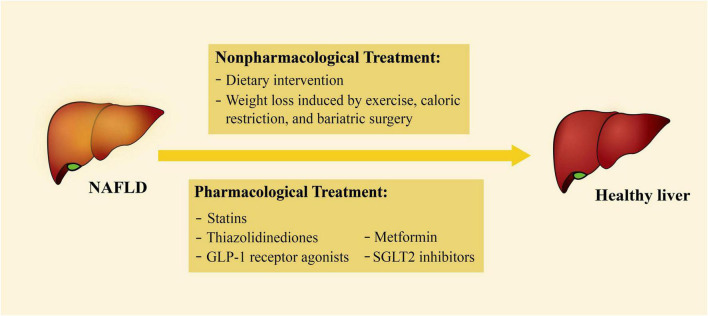
Current treatments for NAFLD. The treatments of NAFLD can be divided into non-pharmacological and pharmacological treatments. Non-pharmacological treatments include dietary intervention, weight loss by exercise, caloric restriction, and bariatric surgery; pharmacological treatments include statins, thiazolidinediones, GLP-1 receptor agonists, SGLT2 inhibitors, and metformin. NAFLD, non-alcoholic fatty liver disease; GLP-1, glucagon-like peptide-1; SGLT2, sodium-glucose cotransporter 2.

At present, there is no specific drug for the treatment of NAFLD. Instead, some non-specific non-pharmacological and pharmacological treatments are currently applied (Figure 2). Some of the treatments have been documented to play a vital role in controlling common risk factors, reducing the effects of secreted factors, or decreasing EAT accumulation. Therefore, these treatments may also have a therapeutic impact on HFpEF (Table 1).

**TABLE 1:** Summary of treatments for NAFLD and their impact on HFpEF.

Intervention	Impact on NAFLD	Impact on common risk factors, secretory factors, and EAT	Impact on HFpEF
Dietary intervention	Improve liver steatosis and fibrosis	Protect against diabetes and obesity	Dietary Intervention is associated with favorable prognosis of patients with HFpEF
Weight loss induced by exercise, caloric restriction, and bariatric surgery	Amelioration of hepatic steatosis or fibrosis	Reduce the risk of diabetes and improve insulin resistance; decrease levels of Inflammatory factors and EAT thickness	Improve cardiorespiratory fitness and exercise capacity
Statins	Improve histological liver damage in individuals with NAFLD/NASH	Have beneficial effects of anti-inflammation and reducing EAT	Reduce mortality rates of patients with HFpEF
Thiazolidinediones	Decrease hepatic inflammation, steatosis, fibrosis	Protect against diabetes; reduce inflammatory markers and alleviate the inflammation of EAT	Delay the development of HFpEF in animal models but lead to sodium retention in patients
Glucagon-like peptide-1 receptor agonists	Reduce the levels of liver enzymes and improved the NAFLD activity score	Lower blood glucose levels and body weight; have anti-inflammatory effects	Alleviate cardiometabolic dysfunction and improve cardiac function in animal models but lack of clinical trials
Sodium-glucose cotransporter 2 inhibitors	Improve steatosis and histological fibrosis; reduce liver fat and the levels of liver enzymes	Improve blood glucose and insulin resistance; decrease epicardial fat volume and CRP	Improvement in left ventricular diastolic function and cardiovascular outcomes in patients with HFpEF
Metformin	Fat accumulation and histological lesions in the liver are ameliorated; decrease the levels of aminotransaminase	Reduce EAT and have the effects of anti-inflammation	Improve clinical outcomes in patients with HFpEF but lack of evidence

NAFLD, non-alcoholic fatty liver disease; EAT, epicardial adipose tissue; HFpEF, heart failure with preserved ejection fraction; NASH, non-alcoholic steatohepatitis; CRP, C-reactive protein.

### 3.1. The influence of non-pharmacological treatment against NAFLD on the development of HFpEF

Non-pharmacological treatment is the cornerstone of NAFLD treatment, which mainly includes dietary intervention and weight loss induced by exercise, caloric restriction, and bariatric surgery. These non-pharmacological approaches are effective in the treatment of NAFLD and also showed promising results in delaying the development of HFpEF.

#### 3.1.1. Dietary intervention

A large number of NAFLD patients overconsume fructose and saturated fatty acids and have reduced intake of dietary fiber and omega-3-rich food (89, 90). Obesity and metabolic syndrome can be caused by such dietary patterns, increasing the risk for NAFLD. Therefore, changes in diet with or without calorie restriction may be a viable and sustainable strategy for the treatment of NAFLD.

The Mediterranean diet, characterized by a large consumption of plant foods, consists of vegetables and fruits, fish, whole grains, legumes, etc. (91, 92). This diet is rich in monounsaturated fatty acids, mainly from low-fat dairy products. The potential association between the Mediterranean diet and the alleviation of NAFLD has been extensively studied. In a randomized, crossover 6-week dietary intervention study, the Mediterranean diet reduced liver steatosis and improved insulin sensitivity among subjects with biopsy-proven NAFLD, even without weight loss (93). At a ten-year follow-up visit, adherence to the Mediterranean diet has been inversely associated with liver steatosis and liver fibrosis (94). In addition to being favorable for NAFLD, the Mediterranean diet improves insulin sensitivity and protects against diabetes and CVD (93, 94). Moreover, it plays a very positive role in the prevention of hypertension and obesity (95, 96). Diabetes mellitus and obesity are the common risk factors for NAFLD and HFpEF. More importantly, in a prospective study, the Mediterranean diet positively affected the prognosis of patients with HFpEF, especially after a 10-year intervention (97). In this prospective study, the Mediterranean diet prevents the recurrence of coronary artery disease by directly ameliorating the effects of inflammation in patients with HFpEF (97). The previous cross-sectional study has also indicated that adherence to a Mediterranean diet can decrease the levels of heart failure biomarkers such as N-terminal pro-brain natriuretic peptide in individuals at high risk of CVD (98). It has also been reported that the Mediterranean diet is beneficial to diastolic and systolic function of the heart in individuals with chronic heart failure (99). Therefore, the Mediterranean diet may have a favorable impact on clinical outcomes in patients with NAFLD and/or HFpEF and should be recommended to these patients.

#### 3.1.2. Weight loss induced by exercise, caloric restriction, and bariatric surgery

Excessive calorie intake and lack of exercise, which lead to obesity and related comorbidities, play a vital role in the development of NAFLD and NASH (100). Lifestyle changes, including weight loss by exercise and caloric restriction, remain the primary treatment for NAFLD management (101).

Weight loss caused by exercise and caloric restriction has a dose-dependent treatment effect on the liver histology of NAFLD patients. Patients with ≥ 5% of total body weight loss can have an improvement in hepatic steatosis, and patients with ≥ 7% of total body weight loss can have significant improvement in steatosis and NAFLD activity score (102). More importantly, among those with ≥ 10% of total body weight loss, liver fibrosis will even regress at least 1 stage (102). The therapeutic effect of weight loss is encouraging in NAFLD. Some common risk factors for NAFLD and HFpEF are also affected by weight loss, such as T2DM (103, 104). More importantly, weight loss caused by exercise and caloric restriction has an impact on EAT and inflammation. In a low-calorie diet weight loss clinical trial, a 6-month low-calorie diet decreased EAT thickness and improved left ventricular mass and diastolic function (105). Inflammatory markers, such as TNFα and CRP, were also significantly decreased by aerobic interval training and a low-calorie diet in a one-year randomized controlled trial (106). In addition, weight loss by exercise and caloric restriction is associated with improvements in cardiorespiratory fitness and exercise capacity among patients with HFpEF (107, 108). These may improve the physical condition of patients with HFpEF and their quality of life. Therefore, weight loss by exercise and a low-calorie diet is highly recommended for patients with NAFLD and may greatly decrease the risk of HFpEF.

The weight loss caused by exercise and caloric restriction has few side effects, but a majority of patients cannot achieve their weight loss goal through exercise and caloric restriction (109). At this time, bariatric surgery can help patients reach this goal. The effects of weight loss caused by bariatric surgery or lifestyle changes seem to be similar for NAFLD, and surgery is more drastic than lifestyle therapies (24). Weight loss caused by bariatric surgery can also alleviate systemic inflammation and control some shared risk factors for NAFLD and HFpEF (110, 111). However, there are conflicting reports in the literature regarding whether bariatric surgery can alleviate EAT. Gaborit et al. reported that epicardial fat decreased significantly after six months of bariatric surgery (112). Nevertheless, Foppa et al. reported that bariatric surgery can significantly decrease subcutaneous fat thickness instead of EAT (113). Moreover, only patients with a BMI > 35 kg/m^2^ can meet the indications for bariatric surgery (114). Therefore, bariatric surgery is not suitable for a majority of people, although it may be a promising treatment for patients with NAFLD and reduce the risk of HFpEF.

Weight loss induced by exercise, caloric restriction, and bariatric surgery has obvious benefits for patients. However, it is difficult to achieve the goal of weight loss through exercise and caloric restriction. In addition, due to strict indications for bariatric surgery, bariatric surgery cannot be applied to people with normal/lean weight. Therefore, some drugs may be needed during the treatment.

### 3.2. The influence of pharmacological treatment against NAFLD on the development of HFpEF

Although there are no drugs approved by the Food and Drug Administration for NAFLD, some non-specific pharmacological treatments currently have shown some benefits in NAFLD such as statins, thiazolidinediones, glucose-like peptide-1 receptor agonists, sodium-glucose cotransporter 2 inhibitors, and metformin. These drugs also have an impact on common risk factors for NAFLD and HFpEF, inflammation, and EAT. Therefore, these drugs may also be beneficial for delaying the development of HFpEF.

#### 3.2.1. Statins

Because metabolic syndrome is related to NAFLD, abnormal lipid metabolism is common in individuals with NAFLD and can increase the risk of CVD (115–117). Therefore, lipid-lowering strategies are critical for individuals with NAFLD. Fortunately, statins can manage the metabolism of lipids such as lowering total cholesterol and low-density lipoprotein.

Statins, hydroxymethylglutaryl-CoA reductase inhibitors, are widely used in various chronic diseases including NAFLD (118, 119). In a multicenter cohort of 1,201 subjects, statins improved histological liver damage in individuals with NAFLD/NASH in a dose-dependent manner and protected against steatosis, steatohepatitis, and fibrosis progression (120). Moreover, statins can increase the therapeutic effect of other drugs on NAFLD by controlling the lipid profile (121). In addition to managing low-density lipoprotein cholesterol, statins have also shown other effects. A randomized controlled trial of atorvastatin and pravastatin therapy reported that statins can also lead to induced EAT regression in patients with hyperlipidemia (122). Furthermore, in other clinical trials, statins have also shown anti-inflammatory effects and alleviated systemic inflammation (28, 123), which will reduce the risk of HFpEF. More importantly, it is reported that statin use is related to improved survival rates in individuals with HFpEF (124, 125). Nochioka et al. also reported that statin use is associated with a better prognosis in HFpEF, independent of the presence of coronary artery disease (124). Statins can also prevent extracellular remodeling, delay the progression of cardiac hypertrophy, improve cardiac diastolic function, and reduce oxidative stress in animal models (126, 127). These findings show the promising results of statins in HFpEF.

However, statins have some side effects. In a randomized, double-blind trial, statins increased the prevalence of diabetes (128), which is supposed to increase the risk of HFpEF. Although statins have been proven to alleviate insulin resistance in animal models, it has been shown in clinical trials that statins can increase the risk of T2DM (129, 130). This may be because statins can impair insulin sensitivity and secretion by pancreatic β-cells and increase insulin resistance in peripheral tissues (131). Although this may cause concern about triggering T2DM, current literature indicates that statins have more advantages than disadvantages in treating NAFLD and reducing the risk of HFpEF. Of course, more studies on the association between statin therapy and the risk of T2DM are needed.

#### 3.2.2. Thiazolidinediones

Peroxisome proliferator-activated receptor-γ (PPAR-γ), a member of the nuclear hormone receptor superfamily, is mainly expressed in adipose tissue. When PPAR-γ is activated, it can enhance lipogenesis, insulin sensitivity, and adipocyte differentiation (132). However, PPAR-γ inactivation in adipose tissue can lead to insulin resistance, metabolic disorders, and excessive fat accumulation in the liver (133). Therefore, PPAR-γ agonists are promising drugs for the treatment of NAFLD. Thiazolidinediones, PPAR-γ agonists, are primarily used to treat diabetes by increasing insulin sensitivity and have also achieved effective results in the treatment of NAFLD in several trials (134–136).

Pioglitazone, a thiazolidinedione, has been investigated in multiple trials. In a randomized, double-blind, placebo-controlled trial, 101 patients with NASH and prediabetes or T2DM received pioglitazone 45 mg/d or placebo for 18 months, followed by pioglitazone treatment for 18 months (134). Pioglitazone significantly improved liver histology including fibrosis in patients with NASH (134). Even low-dose pioglitazone (15 mg/day) can improve hepatic inflammation, steatosis, and insulin resistance in patients in the trial. In addition to the encouraging results in NAFLD treatment, pioglitazone also showed the effects of reducing biomarkers of systemic inflammation and reducing the inflammation of EAT. In a clinical trial of 73 patients with CVD and metabolic syndrome, EAT and plasma inflammatory markers were greatly reduced by pioglitazone, such as IL-6, TNF-α, resistin, and metalloproteinase-9 (137). Furthermore, the expression of proinflammatory and anti-inflammatory genes is reduced in EAT after pioglitazone treatment (138). Pioglitazone can also delay the development of left ventricular fibrosis and HFpEF in animal models (139). Therefore, from these aspects, pioglitazone may reduce the risk of HFpEF.

However, thiazolidinediones can lead to sodium retention and weight gain through anti-natriuretic effects on human proximal tubular cells. Thiazolidinediones can increase the risk of HF, which may be HFpEF or HFrEF (140). Nonetheless, in an Insulin Resistance Intervention After Stroke trial, the study indicated that thiazolidinediones did not increase the risk of developing HF (141). This may be because the action of alleviating systemic inflammation, reducing the secretion of inflammatory biomarkers of EAT, and reducing risk factors for HF by thiazolidinediones dwarfs the side effects of sodium retention, resulting in a neutral or favorable role of thiazolidinediones in HF. In summary, because the effect of thiazolidinediones on HFpEF is still unclear, whether thiazolidinediones can reduce the risk of HFpEF also requires further systemic studies.

#### 3.2.3. Glucagon-like peptide-1 receptor agonists

Glucagon-like peptide-1 (GLP-1) is a hormone produced mainly by intestinal L cells and is an incretin. GLP-1, which is secreted in response to food digestion, stimulates insulin secretion by pancreatic β cells and inhibits glucagon secretion by α cells (142). GLP-1 analogs can lower blood sugar levels and body weight by regulating insulin secretion and suppressing appetite (143). Therefore, GLP-1 receptor agonists play a role in the treatment of NAFLD patients, especially those with T2DM and obesity.

Liraglutide, a long-acting GLP-1 receptor agonist, has shown beneficial effects on NAFLD/NASH in clinical trials. In a multicenter, randomized, double-blind, placebo-controlled trial, liraglutide decreased the levels of liver enzymes and improved the NAFLD activity score which included liver ballooning and lobular inflammation. (144). Furthermore, treatment with liraglutide, taking 1.2 mg daily for 6 months, significantly reduced liver fat content and body weight in patients (145). Similar results are also found in other GLP-1 receptor agonists, such as exenatide (146). The therapeutic effects of GLP-1 receptor agonists on NAFLD are very encouraging. More importantly, GLP-1 receptor agonists show anti-inflammatory effects in preclinical studies and clinical trials. GLP-1 receptor agonists have anti-inflammatory effects on the cardiovascular system, liver, pancreatic islets, and brain by reducing the production of inflammatory markers (147–150). Furthermore, GLP-1 receptor agonists reduce the levels of IL-6 and alleviate oxidative stress and endothelial dysfunction in patients with T2DM or type 1 diabetes mellitus in several clinical trials, which may reduce the risk of HFpEF (151–153). Although GLP-1 receptor agonists have not been invested in patients with HFpEF so far, GLP-1 receptor agonists alleviated cardiometabolic disorders and improved heart function in animal models (154). However, reports on the effects of GLP-1 receptor agonists on EAT are somewhat contradictory. In a clinical trial, 24 participants were randomized to receive liraglutide, and 26 were randomized to receive a placebo (155). Although liraglutide caused the historical resolution of NASH, liraglutide failed to reduce epicardial, hepatic, or myocardial fat. In addition, liraglutide even led to an increase in inflammatory factors in adipose tissue, such as TNF-α and macrophage chemoattractant protein-1 (MCP-1) (156). In contrast, Gianluca Iacobellis et al. reported a substantial and rapid EAT reduction caused by liraglutide (29). Therefore, the effect of GLP-1 receptor agonists on EAT needs further studies.

In summary, GLP-1 receptor agonists show encouraging results in NAFLD and may reduce the risk of HFpEF in patients by improving insulin sensitivity, anti-inflammation, and improving cardiac function. Therefore, GLP-1 receptor agonists deserve further investigation in patients with HFpEF.

#### 3.2.4. Sodium-glucose cotransporter 2 inhibitors

Sodium-glucose cotransporter 2 (SGLT2) inhibitors inhibit the reabsorption of glucose in the proximal tubule of the kidney. SGLT2 inhibitors increase the excretion of glucose, thereby reducing blood glucose and improving insulin resistance (157). The improvement in hyperglycemia and insulin resistance may be related to the reduction of fatty acid synthesis and the blockage of *de novo* lipogenesis (158, 159). Therefore, SGLT2 inhibitors have promising therapeutic potential for the treatment of NAFLD.

Empagliflozin, an SGLT2 inhibitor, is an effective oral antidiabetic drug and has been explored for the treatment of NAFLD. In a clinical trial, NAFLD participants were randomly assigned to empagliflozin (10 mg/day) (N = 43) or placebo (N = 47) for 24 weeks (160). The result is that empagliflozin reduces liver fat and improves steatosis and histological fibrosis, regardless of the presence or absence of diabetes. In another clinical trial, after 20 weeks of empagliflozin treatment, liver fat and the levels of alanine transaminase were decreased in NAFLD patients (161). Moreover, SGLT2 inhibitors (dapagliflozin and luseogliflozin) can also reduce EAT accumulation and alleviate EAT inflammation, which would reduce the risk of HFpEF. During six months of dapagliflozin treatment in a clinical trial, dapagliflozin decreases the EAT volume in patients with diabetes mellitus (162). After 12 weeks of treatment, luseogliflozin can not only improve blood glucose and insulin resistance but also effectively reduce epicardial fat volume and the levels of CRP (163). SGLT2 inhibitors have also been reported to improve left ventricular diastolic function and cardiovascular outcomes such as lowering the risk of adverse events in patients with HFpEF (164–166). Most importantly, in 2022, SGLT2 inhibitors have been approved for the treatment of HF by the American Heart Association/American College of Cardiology (167). In summary, patients with NAFLD and/or HFpEF will benefit from SGLT2 inhibitors treatment.

#### 3.2.5. Metformin

Metformin, a biguanide drug, is a first-line antidiabetic medicine that can improve insulin sensitivity, and regulate the utilization of glucose by the liver. Moreover, metformin has additional effects on metabolism, such as improvements in hepatic gluconeogenesis and fatty acid metabolism (168, 169). Growing evidence indicated that metformin has shown benefits against NAFLD.

In a clinical trial, NAFLD patients were treated with metformin (2000 mg/day) for 48 weeks (170). Liver biopsy, imaging studies, and metabolic profiling were performed at the beginning and end of treatment. The result is that among 30% of NASH patients, liver histology and the level of liver enzymes are improved by metformin. In another randomized trial, metformin reduced aminotransaminase and fat accumulation in the liver and improved histological lesions including fibrosis (171). Furthermore, metformin has the effects of reducing EAT and anti-inflammation, which may decrease the risk of HFpEF. In a double-blind placebo-controlled trial, metformin showed anti-inflammatory effects regardless of diabetes status (172). In clinical settings, metformin can also decrease EAT and reduce adipose tissue inflammation (173, 174). Most importantly, it is reported that the mortality of patients with HFpEF is reduced when treated with metformin (175). However, metformin has also been reported not to be associated with clinical outcomes in patients with HFpEF (176). Accordingly, although metformin shows potential therapeutic effects on NAFLD and HFpEF, the long-term clinical effect of metformin on HFpEF needs further study.

## 4. Conclusion and perspectives

Individuals with NAFLD account for approximately a quarter of the world’s population. Individuals with NAFLD have a higher risk of CVD such as HFpEF. Common risk factors, thicker EAT, and release of secretory factors play an important role in the close association between NAFLD and HFpEF. Hence, the treatments of NAFLD, being able to control common risk factors, decrease the effects of secretory factors, or reduce EAT accumulation, should also have a therapeutic effect on HFpEF.

This review summarizes the major non-pharmacologic and pharmacologic strategies currently applied in NAFLD and their impact on the development of HFpEF. In addition to showing encouraging results in NAFLD, non-pharmacologic therapies can also protect against obesity and diabetes, improve systemic inflammation, and decrease EAT accumulation. Non-pharmacologic therapies can even directly improve the condition of patients with HFpEF. Some drugs also show the potential effects of reducing the risk of HFpEF while showing a therapeutic effect on NAFLD, such as statins, GLP-1 receptor agonists, SGLT2 inhibitors, and metformin. However, some drugs have uncertain effects on the risk of HFpEF. Therefore, the impact of these drugs such as thiazolidinediones on the development of HFpEF needs to be rigorously assessed in subsequent clinical trials.

Although there is a close relationship between NAFLD and HFpEF, the potential mechanism between NAFLD and HFpEF is still unclear and needs to be extensively studied. HFpEF is a systemic disease and the potential mechanisms of HFpEF itself have not been fully clarified so far. Based on a clearer understanding of the mechanism underlying HFpEF, it can be easier to deduce which NAFLD drugs have therapeutic effects on HFpEF. More importantly, uncovering the mechanism of HFpEF should also boost the development of new drugs for HFpEF. In addition, some drugs, such as GLP-1 receptor agonists, have shown encouraging results in preclinical studies. Therefore, authoritative clinical trial studies are needed to further verify whether these drugs have positive effects on HFpEF. Last but not least, as the mechanisms underlying both NAFLD and HFpEF are complex and may vary between different persons, personalized therapies should be applied to different populations.

## Author contributions

ZY and RT wrote the original manuscript. X-JZ, JC, HL, and Z-GS critically reviewed the manuscript. All authors contributed to the article and approved the submitted version.
